# Supplementary material for: Effect of Homochirality of Dipeptide to Polymers’ Degradation
Source: Polymers (Basel). 2020 Sep 22;12(9):2164. doi: 10.3390/polym12092164 (PMC7570312; doi:10.3390/polym12092164)
Supplement: Supplementary file 1 [file polymers-12-02164-s001.pdf]

Supplementary Material

Effect of homochirality of dipeptide to polymers' degradation

Xinqiang Xu [1,2], Fuyan He [1,2], Wenke Yang [1,2] and Jinshui Yao [1,2*]

[1] School of Materials Science & Engineering, Qilu University of Technology (Shandong Academy of Sciences), Jinan 250353, China; xu_xin_qiang@163.com (X.X.); hefuyan555@163.com (F.H.); wkyang@qlu.edu.cn (W.Y.)
[2] Shandong Provincial Key Laboratory of Processing & Testing Technology of Glass and Functional Ceramics, Jinan 250353, China
* Correspondence: yaojsh@qlu.edu.cn

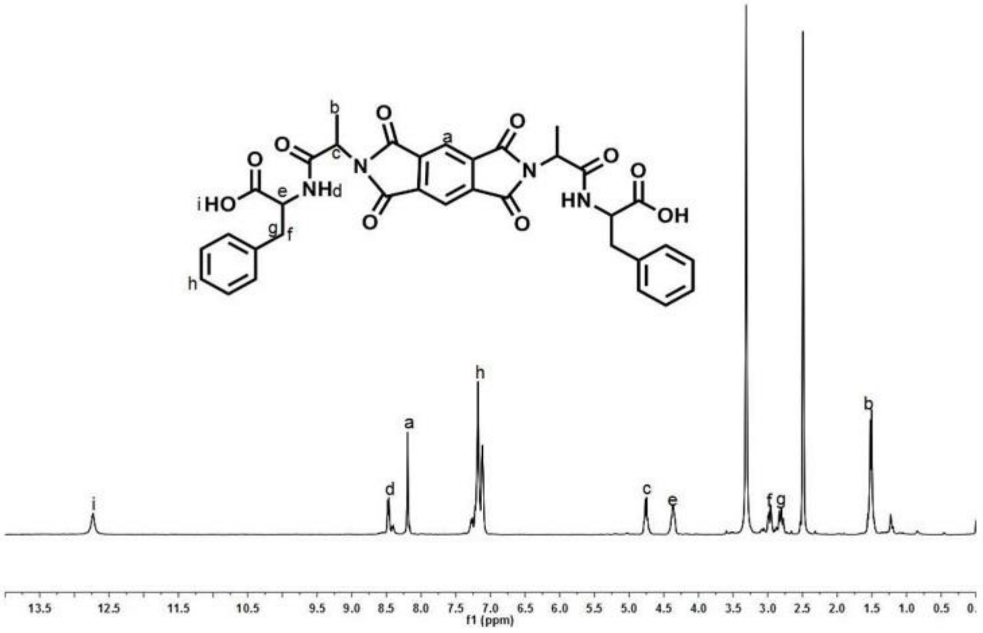

Figure S1: Representative ^{1}H NMR spectrum of monomers.

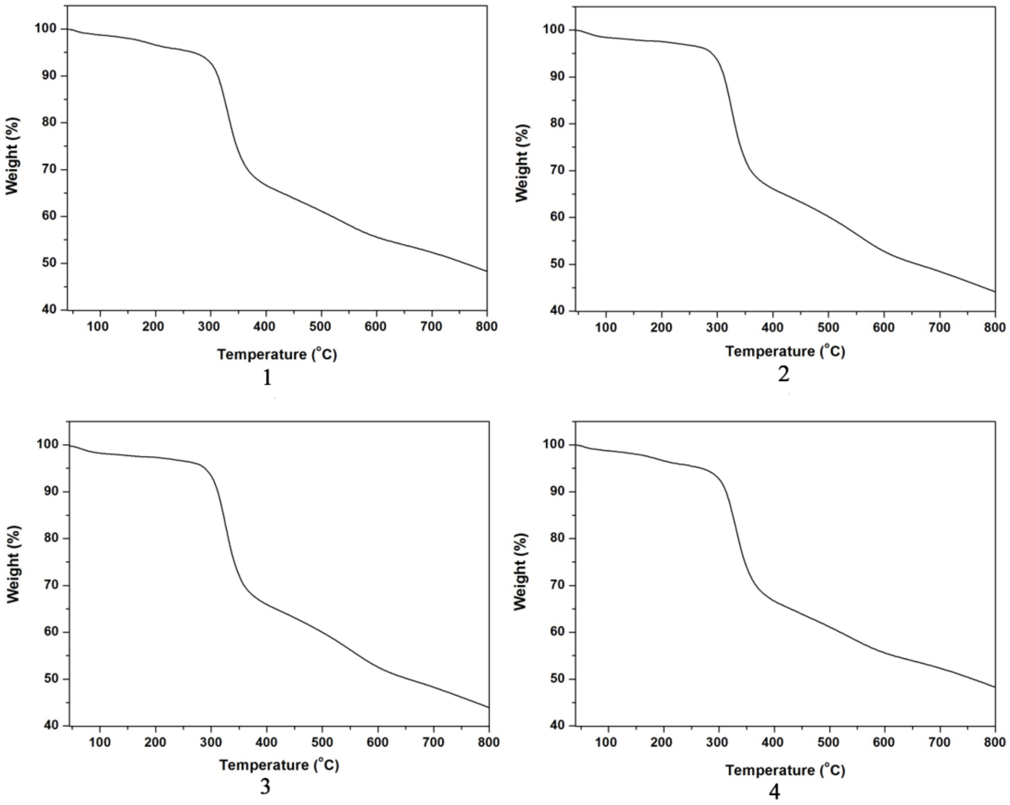

Figure S2: TGA graphs of PAI 1-4.

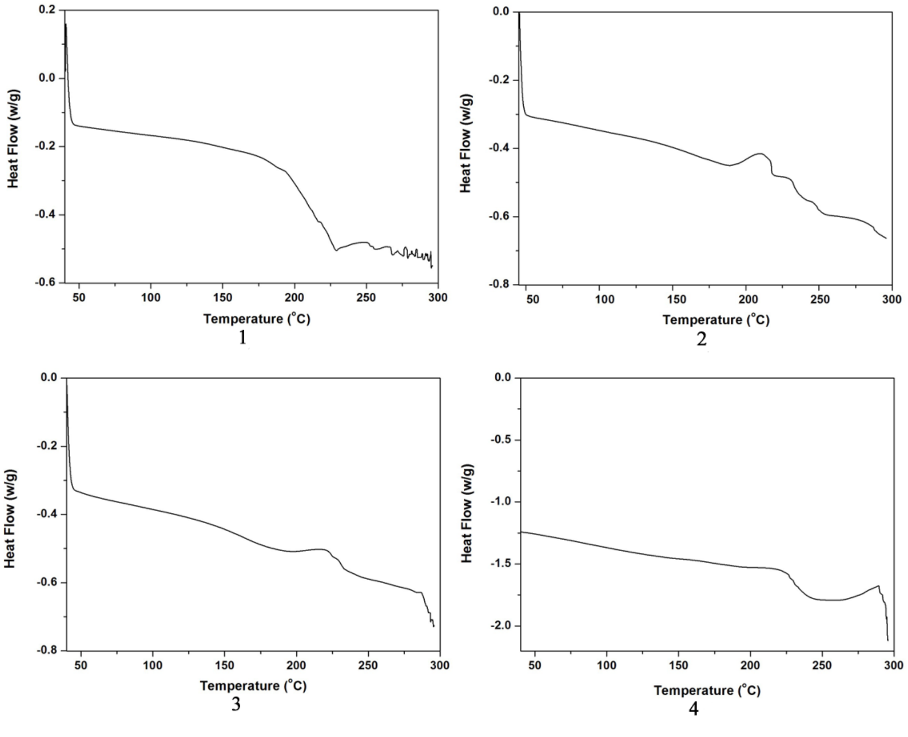

Figure S3: DSC graphs of PAI 1-4.

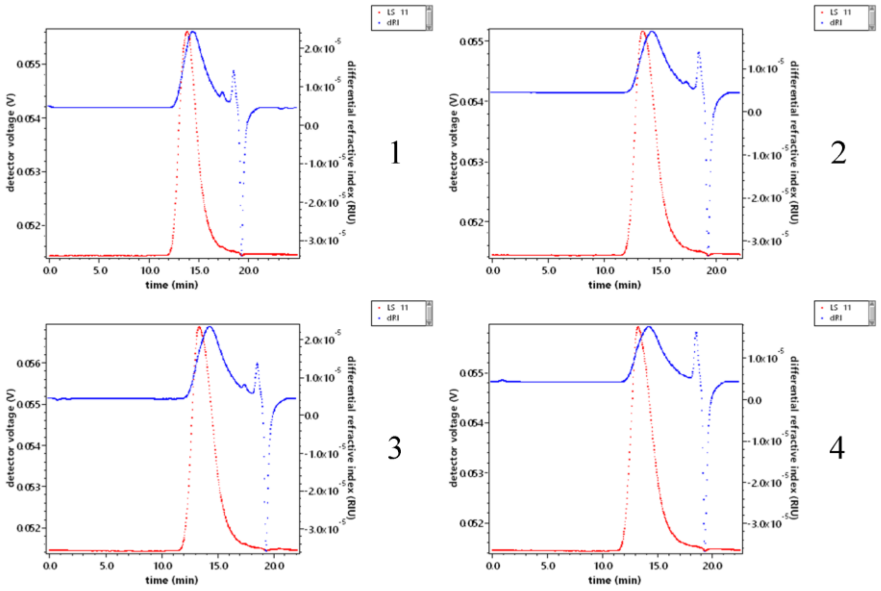

Figure S4: GPC graphs of PAI 1-4 after degradation.
